# How do local government information sources affect the purchase willingness of low-carbon agricultural products? The example of regional brand agricultural products

**DOI:** 10.3389/fpubh.2023.1169741

**Published:** 2023-03-28

**Authors:** Pu Xu, Linlin Wang, Yufeng Li, Min Liu

**Affiliations:** ^1^School of Economics and Management, Shanghai Ocean University, Shanghai, China; ^2^School of Humanities and Management, Guilin Medical University, Guangxi, China

**Keywords:** low-carbon agriculture, low-carbon agricultural products, regional brand agricultural products, information source characteristics, perceived benefits, perceived risk

## Abstract

**Introduction:**

In order to achieve the carbon peaking and carbon neutrality goals, the agricultural sector has been given high priority to reduce carbon emissions. Since consumption is the ultimate goal of production, the consumption of low-carbon agricultural products is of great significance to promote the reduction of agricultural carbon emissions. Regional brand agricultural product is an important tool to promote the development of regional economy to “green, low-carbon, branded and high-quality”, and has the technical and institutional conditions to develop into low-carbon agricultural product, so this study takes regional brand agricultural products as the representative of low-carbon agricultural products. As a information source to guide the public to consume in the green and low-carbon way, local government can effectively develop the market for low-carbon agricultural products and drive the development of low-carbon agriculture from the demand side.

**Methods:**

Based on structural equation model with bootstrap method, this paper focuses on the mechanism of the influence of local government information source characteristics (credibility, professionalism, and attractiveness) on consumers' willingness to purchase low-carbon agricultural products, and explores the mediating role of perceived benefits and perceived risks.

**Results:**

The following findings are established: first, the credibility and professionalism of local governments play a positive role in influencing the purchase willingness of low-carbon agricultural products through perceived benefits, with credibility having the greatest degree of influence. Second, the attractiveness of local governments positively influences consumers' willingness to purchase low-carbon agricultural products through perceived risk. Third, perceived benefits play a fully mediating role between credibility and purchase intention, play a partially mediating role between professionalism and purchase willingness, perceived risks play a partially mediating role between attractiveness and purchase willingness.

**Discussion:**

This study provides new ideas for the construction of low-carbon agricultural products and low-carbon development in the agricultural sector from the perspective of local government information sources.

## 1. Introduction

Global warming poses a serious threat to the ecological environment, human health and sustainable economic and social development (1), China committed the “double carbon” target to the international community in September 2020. Human activities are a major source of carbon emissions, with the agricultural sector accounting for 17% of global emissions (2), so the issue of low-carbon emissions reduction in agriculture has received widespread attention. In August 2021, China's Ministry of Agriculture and Rural Affairs issued the “14^th^ Five-Year Plan for National Agricultural Green Development”, stating that it would “accelerate the establishment of a green, low-carbon and recycling agricultural industry system”. Academics have conducted research on the basic status, potential assessment, and drivers of low-carbon development in agriculture from a production perspective (3, 4), but less attention has been paid to the demand perspective to study the driving role of low-carbon agricultural consumption on low-carbon agricultural development.

Low-carbon agricultural products refer to agricultural products with carbon labels (5), carbon labeling is an important information disclosure tool that can reflect the carbon emissions of agricultural products, and can be used to record the greenhouse gas emissions of agricultural products during the whole life cycle, such as raw material procurement, manufacturing and processing, logistics and transportation, consumption and waste recycling and disposal, etc., and then convert them into carbon dioxide equivalent and inform consumers in the form of labels (6, 7). The establishment of China's carbon labeling system is still in its initial stage, and there are still institutional and technical limitations to the popularization of low-carbon agricultural products. Regional public brand agricultural products (later referred to as regional brand agricultural products) have the potential to develop into low-carbon agricultural products, and it is undoubtedly more efficient to realize low-carbon agricultural development with regional brand agricultural products as the carrier. From the production side, due to the limitations of scale, capital, technology and management ability, it is difficult for new agricultural business entities to independently produce and sell low-carbon agricultural products. In contrast, regional brand built by local governments as the main guide, industry associations, leading enterprises, cooperatives, farmers, scientific research institutions, and other common construction is an important way to promote the regional economy to “green, low-carbon, brand, high-quality” development. Low-carbon agricultural products are the goal of the development of regional brand agricultural products, and regional brand can take the region as a whole unit, gather the power of each stakeholder in the industry, select the type of agricultural products according to the local environment and ecological endowment, and gradually achieve the low-carbon technology application, low-carbon planting, low-carbon processing, low-carbon transportation, and low-carbon sales and other whole process to be low-carbon so as to promote low-carbon agricultural development under the leadership of the local government. From the consumer side, most of the regional brands of agricultural products with characteristics have already gained a certain market popularity after years of development. As a kind of label, regional brand agricultural products can deliver reliable quality signals to consumers, weakening the shortcomings of the diversity of categories and hidden quality of agricultural products themselves. While low-carbon agricultural products, as an emerging label for agricultural products, can deliver reliable quality signals in addition to the concept of green consumption. Relying on the existing regional brands to further develop low-carbon agricultural products, we can not only use the popularity of regional brands to quickly open the market, but also make low-carbon agricultural products more easily accepted by consumers.

Academics have focused on low-carbon agricultural products from the consumer side and discussed them from the perspectives of willingness, attitude, and perception. In addition to the quality of agricultural products, image of origin, and promotional media (8, 9), consumers' willingness to purchase is also influenced by information sources. In this paper, local government is studied as an important information source, on the one hand, it is because the government's information participation plays an important role in enhancing consumer confidence and guiding consumers to establish the concept of green consumption. Due to information asymmetry, consumers in an information disadvantageous position will try to obtain more information from the government, media and other third parties (10), the local government as a reliable information source can transmit signals of reliable quality and guaranteed safety when promoting low-carbon agricultural products, which can effectively solve the problems of information asymmetry and consumers' difficulty in accessing high-quality information. On the other hand, as the main guide and strong engine for the development of low-carbon agricultural products and regional brand agricultural products (11), the local government focuses on the construction of governance system, and the actual information participation behavior is still insufficient. The connection between government and consumers is still loose, the process and effect of information participation are not effectively delivered to the consumer side to enhance the value of low-carbon agricultural products. In addition, studies on government participation in the literature have focused on policy formulation, resource integration, industrial subsidies, and market supervision (12, 13), but there is a lack of research on the impact on consumers' purchase intention from the perspective of information participation, using local governments as information sources.

This paper takes regional brand agricultural products as a representative of low-carbon agricultural products and analyzes the intrinsic mechanism of information source characteristics affecting consumers' willingness to purchase regional brand agricultural products. Perceived benefit-perceived risk is introduced to propose a theoretical framework that the characteristics of local government information sources influence consumers' perceived benefit and perceived risk and thus their willingness to purchase, and finally, the proposed conceptual model and research hypotheses are tested through questionnaire research.

## 2. Literature review

### 2.1. Research on the development status of low carbon agricultural products

In order to achieve the goal of carbon peaking and carbon neutrality, the carbon emission detection, reporting and verification system is gaining more and more attention, and the carbon information disclosure mechanism established by carbon labeling has come into being (14), the carbon label for agricultural products is a specific application of carbon labeling in the field of agricultural products, and is an important tool for agricultural carbon information disclosure (15). The carbon label for agricultural products is similar to the existing green label in China, which can provide information on the environment of the origin of agricultural products, supplement the producer's behavior information, carbon emission information, etc., and further improve the quality and price system of agricultural products (14). At present, the carbon labeling system of agricultural products in China is still in the exploration stage (14), and studies have shown that the establishment of carbon labeling system requires high human, material and financial resources, and it is difficult to rely on government power alone to promote it. So, Hu Yu and other scholars believe that it is necessary to accelerate the construction of a sustainable carbon labeling system for agricultural products with the active participation of government, enterprises, collectives and farmers (14). The regional brands of agricultural products are created by local governments, industry associations, agricultural enterprises, cooperatives, farmers, and research institutions (16), which have a good working basis for the development of carbon labeling system.

There are studies prove that regional brand agricultural products can be used as the entry point for the construction of carbon labeling system, Hu Yu and other scholars took Hongze District, Jiangsu Province as an example and introduced the mechanism for the construction of carbon labeling system for rice, the carbon labeling certification system for rice constructed in Hongze District took the regional brand “Forked River Rice” as the entry point, and the mechanism for the construction of carbon labeling system involved the collaboration of local government, rural collective economic organizations, farmers and research institutions. Jin et al. (15) and other scholars summarized the carbon label certification in the field of agricultural production. In the field of primary agricultural products, Tianmu fruit shoots in Lin'an, Zhejiang Province, is the first agricultural product with carbon label in Zhejiang Province (15), which also relies on the establishment of regional brand.

Therefore, with the requirement of low carbon development in agriculture, the importance of regional brand agricultural products in the process of carbon labeling system construction will gradually emerge, and it will become a trend to promote the popularization of carbon labeling with regional brand agricultural products as the main way. So, this paper takes regional brand agricultural products as a representative of low-carbon agricultural products and studies the factors that influence consumers' willingness to purchase low-carbon agricultural products.

### 2.2. Research on the factors influencing the purchase intention of low carbon agricultural products and regional brand agricultural products

The factors influencing the purchase intention of low-carbon agricultural products and regional brand agricultural products in existing studies can be divided into internal and external aspects. Internal factors are personal and family characteristics of consumers, such as age, education level, income level, cognitive level, etc., (17). Zhang and Guo concluded that the education level of individuals and families has a significant effect on the intention to consume low-carbon agricultural products (18). Wang and Li found that family size has an influence on consumers' purchase of agricultural products (10). Zhao believes that consumers' cognitive level can influence or change consumers' behavior, and the study proved that knowledge level positively influences consumers' willingness to purchase by negatively affecting perceived risk, she also proved that consumption level can positively influence consumers' willingness to purchase by positively affecting perceived benefits (19). Zhang et al. (20) investigated the consumption of vegetables with carbon labels by Shanghai residents, and found that consumers' awareness of carbon labels significantly influenced purchase intentions. Several scholars have explored the consumption intention of low-carbon agricultural products from the perspectives of healthy development (21), environmental attitudes (22), and environmental values (23).

The external factors include the quality of agricultural products, attributes of agricultural products, price, brand image and culture, brand awareness, sales platform, and publicity media. Many scholars believe that the quality and circulation mode of agricultural products affect consumers' consumption intentions by influencing their perceived value and brand trust (24, 25). Yang believes that brand awareness is one of the important factors affecting the consumption of regional brand agricultural products (26). Wang and Wang divided the regional brand image of agricultural products into three dimensions: regional product or service image, user image, and regional industry image, and constructed a model of regional brand image of agricultural products and consumers' purchase intention (27). They proved that each image dimension of regional brand has positive influence on consumers' purchase intention (27). Lai divided brand stories into weak stories and strong stories, and proved that both have positive influence on consumers' purchase intention, and weak stories have better effect (28).

With the popularity of e-commerce live marketing mode, Zhao (29) explored the influence on consumers' purchase of regional brand agricultural products from the perspective of host characteristics. She concluded that the professional, well-known, interactive and emotional nature of hosts significantly influence the formation of consumer preferences and consumption intentions (29). Using e-commerce anchors as an information source, Zhao (29) investigated the effect of their characteristics on consumers' purchase intention. In addition, studies on the characteristics of information sources as an influencing factor have also yielded some results in the literature on consumer purchase intention. Liu et al. (30) found that the professionalism and trustworthiness of information sources can increase consumption intention by promoting consumption confidence, and Meng et al. (31) investigated the inner mechanism of information source characteristics affecting consumption intention, and concluded that information source characteristics can positively influence consumption intention by influencing consumers' perception paths of emotional dependence and face perception, and by influencing the rational path of relationship norm perception.

In summary, studies have proved that in the process of realizing low-carbon agricultural development, regional brand agricultural products have become an important entry point, but there is still a lack of research on low-carbon agricultural development driven by the consumer perspective of regional brand agricultural products. The research on the factors influencing consumers' willingness to purchase low-carbon agricultural products is mostly focused on consumers' personal characteristics and attributes of agricultural products, brand characteristics, platform characteristics, etc., but there isn't enough attention paid to the participation behavior of industrial stakeholders, such as local governments, industry associations and cooperatives in the process of building regional brand and developing low-carbon agriculture. Therefore, this paper further expands on the existing theoretical basis: it focuses on the local government, which plays a leading role in the promotion and production of low-carbon agricultural products, regards the local government as the source of information for consumers, and analyzes the influence of the characteristics of local government information sources on consumers' willingness to purchase low-carbon agricultural products.

In this paper, we analyze the underlying mechanisms that influence consumers' willingness to purchase low-carbon agricultural products from the perspective of information source characteristics, focusing on the following questions: Do local government information source characteristics influence consumers' willingness to purchase? In what way and to what extent do the credibility, professionalism and attractiveness of local government play a role in consumers' willingness to purchase? Do consumers' perceived benefits and perceived risks play a role as mediating variables in this process? What is the extent of their influence?

## 3. Research hypothesis and theoretical framework

### 3.1. Research hypothesis

#### 3.1.1. Relationship between information source characteristics and perceived benefits, perceived risks

As a kind of information label, if information source is authoritative and credible enough, can effectively reduce information asymmetry (32), consumers in an information disadvantageous position will try to obtain more information from the government, enterprises, media and other third-party institutions to reduce the disadvantage, and consumers can perceive the utility and value of the product from the information label. So reliable information sources can transmit the safe and guaranteed signals of product quality in an invisible way. There is a significant positive impact on the perceived benefits (10).

Information sources can influence individuals' understanding and perception of product information in terms of three characteristics: credibility, professionalism and attractiveness (31). Credibility refers to the degree of trusting the information source from consumers (33), Endogan believes that the credibility of information sources affects consumers' perceptions of products, and that higher credibility leads to more positive evaluation of products (34) and easier reliance on product brands, Qi and Yang believe that consumers will also give priority to products introduced by their trusted information sources when they have new needs (35). Therefore, when purchasing regional brand agricultural products, consumers believe that the higher the credibility of the local government, the easier it is to make a positive evaluation on the publicity, promotion and products provided by the local government, which can effectively reduce the perceived risks of consumers and enable them to obtain higher expected value in the process of purchase and consumption, thus consumers will have higher perceived benefits. Accordingly, this study proposes the following hypotheses.

H1: The credibility of local government positively affects consumers' perceived benefits.
H2: The credibility of local government negatively affects consumers' perceived risk.

Professionalism refers to the knowledge, experience or skills possessed by the information source that can be referred to (36). Biswas argued that the professionalism of the information source can significantly influence users' risk perceptions and attitudes (37), and that consumers tend to recognize and trust a product when it is promoted by someone with professional knowledge and background (35). Therefore, when consumers buy regional brand agricultural products, the stronger the professionalism of local government, the more adequate its knowledge about agricultural production and cultivation, regional brand construction and operation, the more experienced it is in identifying the authenticity of regional brands, the more qualified it is to promote and publicize regional brands, the higher quality and more persuasive the information output will be. The more effective it will be in enhancing consumers' acceptance, saving the time and energy of information audiences. In other words, professional information sources can reduce consumers' perception of risk and increase their perceived benefits. Accordingly, this study proposes the following hypotheses.

H3:The professionalism of local government positively affects consumers' perceived benefits.
H4: The professionalism of local government negatively affects consumers' perceived risk.

Attractiveness refers to the ability of an information source to attract the attention of the information audience (38), and the information source validity model suggests that the attractiveness of an information source includes familiarity, affection and similarity (39). Attractiveness can arouse the curiosity of information audiences and enhance consumers' attention to information content, and consumers' affection and familiarity with the information source will bring strong perceived value (35). Therefore, when the local government is promoting the regional brand agricultural products, it can follow the development of the times to grab the attention of consumers through new communication methods such as graphic, video and live broadcast, etc. The degree of consumers' affection and familiarity with the local government will be also deepened, in other words attractiveness of local government can increase consumers' perceived benefits. Accordingly, this study proposes the following hypothesis.

H5: The attractiveness of local government positively affects consumers' perceived benefits.
H6: The attractiveness of local government negatively affects consumers' perceived risk.

#### 3.1.2. The relationship between perceived benefits, perceived risks and purchase willingness

In the framework of Perceived benefit—Perceived risk, the higher the perceived benefit and the lower the perceived risk, the more it can positively influence consumers' decisions (40). Regional brand as a quality signal can influence consumers' quality perception of agricultural products and stimulate consumers' associations, such as economic support for local producers (41), and consumers will have higher perceived benefits; and the long-term popularity of regional brand can make consumers have lower perception of risk for after-sales service and other aspects, so consumers' willingness to purchase is stronger. Accordingly, this study proposes the following hypotheses.

H7: Perceived benefits positively affect consumers' willingness to purchase regional brand agricultural products.
H8: Perceived risk negatively affects consumers' willingness to purchase regional brand agricultural products.

### 3.2. Theoretical framework

**Figure 1:**
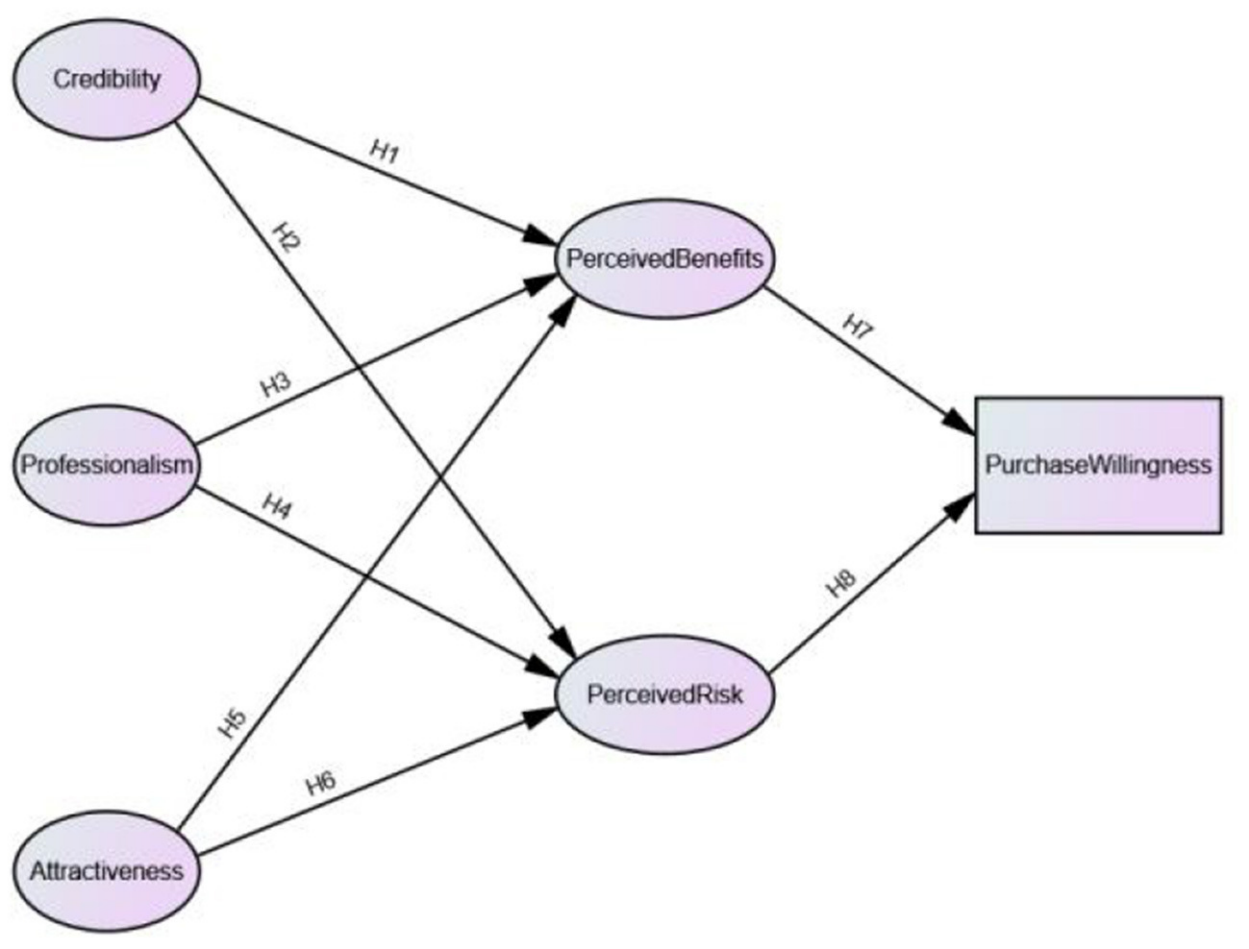
The path model of local government information source characteristics influencing the consumption willingness of regional brand agricultural products.

Based on the above literature theories and research hypotheses, this paper introduces the perceived benefit-perceived risk framework as a mediating variable to construct the mechanism and path of local government information source characteristics affecting willingness to purchase regional brand agricultural products, and the complete theoretical model framework is shown in Figure 1.

## 4. Data and methodology

### 4.1. Data source and variable measurement

#### 4.1.1. Sample selection and data collection

In this study, a random sample of consumers across the country was surveyed using a combination of the Credamo platform and the Questionnaire Star platform. The group issued a total of 356 questionnaires, of which 342 were valid, with an efficiency rate of 96%. From the results of the questionnaire survey, it can be seen that the respondents are mostly female, accounting for 58.5%, the respondents are mostly young, with consumers aged 21–40 accounting for 92.4%, the acquired education is mostly undergraduate, accounting for 70%, most of them are employees of enterprises and institutions, accounting for 75.2%, and the monthly income level is distributed more evenly, the basic situation of the sample is shown in Table 1.

**Table 1:** Respondents' basic characteristics.

**Projects**	**Category**	**Number**	**Proportion**	**Projects**	**Category**	**Number**	**Proportion**
Gender	Male	142	41.5%	Age	Under 20	6	1.8%
	Female	200	58.5%		21–30	197	57.6%
Educational background	Junior high school	13	3.8%		31–40	119	34.8%
	Junior college	35	10.2%		41–50	11	3.2%
	Undergraduate	240	70.2%		51 or above	9	2.6%
	Master and above	54	15.8%	Monthly income (RMB)	Under 3,000	69	20.2%
Career	Students	75	21.9%		3,000–5,000	62	18.1%
	State-owned enterprises/institutions/civil servants	93	27.2%		5,000–8,000	76	22.2%
	Private/foreign enterprise	164	48.0%		8,000–15,000	82	24.0%
	Freelance	10	2.9%		15,000 or more	53	15.5%

#### 4.1.2. Variable measurement

Characteristics of local government information sources: This study classifies the characteristics of local government information sources into credibility, professionalism and attractiveness. Based on the theoretical study and empirical scale of information source characteristics (31), three characteristics are measured as follows: the questions for credibility include “the information source has good credibility, the released information is reliable, the released information is objective, the released information is complete”. The questions for professionalism include “local government has professional knowledge of regional brand agricultural products, has the ability to identify the authenticity of regional brand agricultural products, is qualified to promote regional brand, and is an effective information channel”. The questions for attractiveness include “the released information is attractive; the released information gives people a sense of pleasure; the released information is interesting to me; I like the released information”.

Perceived risk and perceived benefit: Drawing on the scale of perceived value, this study combines the characteristics of regional brand agricultural products, and measures the perceived risk and perceived benefit as follows: The questions for perceived benefit include “regional brand agricultural products have better quality, the brand is easier to remember, the brand has higher social recognition, the brand can promote low carbon development in agriculture”. The questions for perceived risks include “when buying regional brand agricultural products, we worry about buying fake products, we worry about bad taste and flavor, we worry about quality and safety problems, we worry about serious transportation loss, we worry about bad after-sale service”.

Willingness to purchase regional brand agricultural products: In this study, the willingness to consume is used as the explanatory variable. In contrast to the explanatory variables, the credibility, professionalism and attractiveness of local governments need to be reflected from multiple aspects in order to be more scientific, and in contrast to psychological variables that cannot be measured directly, such as attitudes and perceptions, the explanatory variables in this paper refer to existing studies to reflect the purchase intentions of consumers directly through a five-point Likert scale. The question is “How willing are you to buy regional brand agricultural products”, which is divided into 5 levels: “very unwilling, less willing, generally willing, more willing and very willing”.

### 4.2. Reliability and validity of the scale

The measurement items selected for this study were all based on the domestic and international literature, and the questions were designed according to the need to study the consumption willingness of regional brand agricultural products, combined with the judgment of experts in the field, so that the scale has surface validity. The questionnaire was pre-researched in two rounds and fully revised for mass distribution. Table 2 shows the results of the statistical tests of the scale reliability and validity, the scale consistency reliability test is based on the Cronbach's α coefficient, and it can be seen from the table that the Cronbach's α coefficient of each concept in this study is greater than 0.8, which is greater than the critical level of 0.7. The scale validity is used to analyze whether the index items are reasonable and meaningful. In this study, the KMO values initially reflected the validity level of the scale, and the table shows that the KMO of each latent variable was between 0.7 and 0.9, which indicates that each measure is suitable for information extraction and has good validity. The standardized factor loadings of measurements on each concept are greater than 0.6, which is greater than the minimum critical level of 0.60 required by relevant studies, indicating that the questionnaire has good convergent validity. The Average Variance Extracted (AVE) and Composite Reliability (CR) of latent variables are used for convergent validity analysis, and the convergent validity is better when AVE is greater than 0.5, and the AVE of each latent variable in this study is higher than 0.5; CR indicates the combination of the reliability of all observed variables of latent variables, and above 0.7 indicates better combined reliability, and the table shows that the CR of each latent variable in this paper is greater than 0.80, which indicates that the consistency among the observed variables of the latent variables is good.

**Table 2:** Scale reliability, validity, and the confirmatory factor analysis test results.

**Concepts (latent variables) and measures (explicit variables)**	**Cronbach's alpha**	**KMO**	**Standardized factor loadings**	**CR**	**AVE**
**Information source credibility (ISC)**					
(ISC1) local government has good credibility	0.85	0.82 (0.000)	0.79	0.85	0.59
(ISC2) the released information is reliable			0.75		
(ISC3) the released information is objective			0.74		
(ISC4) the released information is complete			0.79		
**Information source professionalism (ISP)**					
(ISP1) has professional knowledge of regional brand agricultural products	0.83	0.77 (0.000)	0.79	0.90	0.72
(ISP2) has the ability to identify the authenticity of regional brand agricultural products			0.83		
(ISP3) is qualified to promote regional brands			0.68		
(ISP4) is an effective information channel			0.71		
**Information Source Attractiveness (ISA)**					
(ISA1) the released information is attractive	0.91	0.85 (0.000)	0.84	0.91	0.57
(ISA2) the released information gives people a sense of pleasure			0.80		
(ISA3) the released information keeps me interested			0.87		
(ISA4) I like the released information			0.87		
**Perceived Benefit (PB)**					
(PB1) regional brand agricultural products have better quality	0.82	0.83 (0.000)	0.75	0.84	0.50
(PB2) the brand is better packaging			0.60		
(PB3) the brand is easier to remember			0.73		
(PB4) the brand has higher social recognition			0.74		
(PB5) the brand can promote low carbon development in agriculture			0.70		
**Perceived Risk (PR)**					
(PR1) when buying regional brand agricultural products, we worry about buying fake products	0.89	0.85 (0.000)	0.82	0.90	0.64
(PR2) we worry about bad taste and flavor			0.86		
(PR3) we worry about quality and safety problems			0.90		
(PR4) we worry about serious transportation loss			0.70		
(PR5) we worry about bad after-sale service			0.72		

The brackets of KMO are the size of the *p*-value.

The recovered data were tested for discriminant validity, and the diagonal lines in the table are the AVE square root values, and the remaining values are the Pearson correlation coefficients. If the square root of each AVE in the model is greater than the correlation coefficient between the concept and the other concepts, then this questionnaire is said to have good discriminant validity (42). From Table 3, the square root of the AVE values on the diagonal of each factor is greater than the maximum value of the absolute value of the correlation coefficient between the factors, indicating that the concepts in this paper have good discriminant validity.

**Table 3:** Discriminant validity: Pearson correlation and AVE square root values.

	**Information source credibility**	**Information source attractiveness**	**Information source professionalism**	**Perceived benefits**	**Perceived risk**
Information source credibility	0.77				
Information source attractiveness	0.59	0.85			
Information source professionalism	0.69	0.60	0.75		
Perceived benefits	0.57	0.54	0.59	0.70	
Perceived risk	−0.11	−0.23	−0.08	−0.24	0.80

## 5. Empirical analysis

### 5.1. Model evaluation

In this study, a structural equation model was constructed using the software AMOS28.0. The model fitted the path of local government information source characteristics influencing the purchase willingness of regional brand agricultural products and was modified on the basis of the initial model, and the test results are shown in Table 4. The absolute fit measures, incremental fit measurement and parsimonious fit measurement all meet the requirements, indicating that the research model has good explanatory power and can be used to test the theoretical hypotheses in the previous section.

**Table 4:** Modified model fitness test values.

**Projects**	**Indicators**	**Threshold**	**Models**	**References**
Absolute fit measurement	χ^2^/df	< 3	1.84	Hair et al. (43)
	GFI	>0.8	0.88	Bagozzi and Yi (44)
	AGFI	>0.8	0.85	Bagozzi and Yi (44)
	RMSEA	< 0.08	0.06	Hair et al. (43)
Incremental fit measurement	IFI	>0.9	0.95	Hair et al. (43)
	CFI	>0.9	0.95	Bagozzi and Yi (44)
	NFI	>0.9	0.90	Bagozzi and Yi (44)
Parsimonious fit measurement	PGFI	>0.5	0.70	Bagozzi and Yi (44)
	PNFI	>0.5	0.78	Hair et al. (43)

### 5.2. Hypothesis testing results

As can be seen from Table 5, the standardized estimated coefficients of credibility and professionalism on perceived benefits are 0.312 and 0.295, which are significant at the 1% level, indicating that hypotheses H1 and H3 are valid, and the degree of influence of credibility on perceived benefits is higher than that of professionalism. On the one hand, this may be due to the fact that the credibility of the local government comes from its long-accumulated reputation and consumers are willing to trust the official endorsement of the local government for regional branded agricultural products due to inertia psychology. On the other hand, local governments play an important role in the whole life cycle of the construction of regional brand of agricultural products. For example, they play the role of advocate and planner in the early stage of construction. After the brand is built, the local governments will be transformed into the supervisor of brand development and the provider of public services, and play the role of support and manager for the subjects involved in brand construction. In the context of the overall leadership of local governments, compared with ordinary agricultural products, consumers are more inclined to trust the quality of regional brand agricultural products, so as to have a higher perception of benefits. Professionalism also has a significant positive impact on consumers' perceived benefits, because the more information released by local governments can demonstrate the expertise in regional brands, such as setting production standards for regional brand agricultural products, providing standards for authenticating brands and providing authentic brand logos, the more consumers can feel the signal of brand quality assurance, and consumers will be more inclined to trust that regional brand agricultural products has higher product quality, thus the consumers' perceived benefits will be increased.

**Table 5:** Standardized path coefficients and hypothesis testing results.

**Paths**	**Standardized estimated coefficients**	**S.E**.	**C.R**.	**P**
Perceived benefits < - credibility	0.312	0.104	2.994	**
Perceived benefits < -professionalism	0.295	0.099	2.968	**
Perceived risk < -attractiveness	−0.353	0.087	−4.047	***
Purchase willingness < - perceived benefits	0.771	0.094	8.222	***
Purchase willingness < - perceived risk	−0.098	0.041	−2.406	**

***represents significant at the 1% level, **represents significant at the 5% level.

The standardized estimated coefficient of local government attractiveness on perceived risk is −0.353 and is significant at the 0.1% level, indicating that hypothesis H6 is valid, the effects of credibility and professionalism on perceived risk do not pass the significance test, indicating that hypotheses H2 and H4 are not valid. The source validity model considers familiarity, affection and similarity together as the source attractiveness, with the development of the Internet, the channels of government information release are gradually diversified, and consumers will receive information about regional brand agricultural products released by local government through various ways such as graphic, short video and live broadcast, etc. The higher the familiarity and fondness of consumers with local government, the more they will think that regional brands promoted by local governments have lower quality and safety risks, and regional brand agricultural products purchased from official government platforms will have better service guarantees, thus significantly reducing consumers' perceived risks.

As shown in Table 5, the standardized estimated coefficients of perceived benefits and perceived risks on purchase willingness are 0.771 and −0.098, which are significant at 0.1 and 5% levels, indicating that hypotheses H7 and H8 are valid. Because the higher the perceived benefits and the lower the perceived risks, the more consumers can obtain the expected utility in the process of consuming regional brand agricultural products. It can be seen from the comparison of the standardized coefficients that the influence of perceived benefits is greater than the influence of perceived risks. In other words, consumers are more concerned about the actual benefits that regional brand agricultural products can bring in the purchase process, such as higher quality, better taste, and more comprehensive after-sales service of agricultural products.

**Figure 2:**
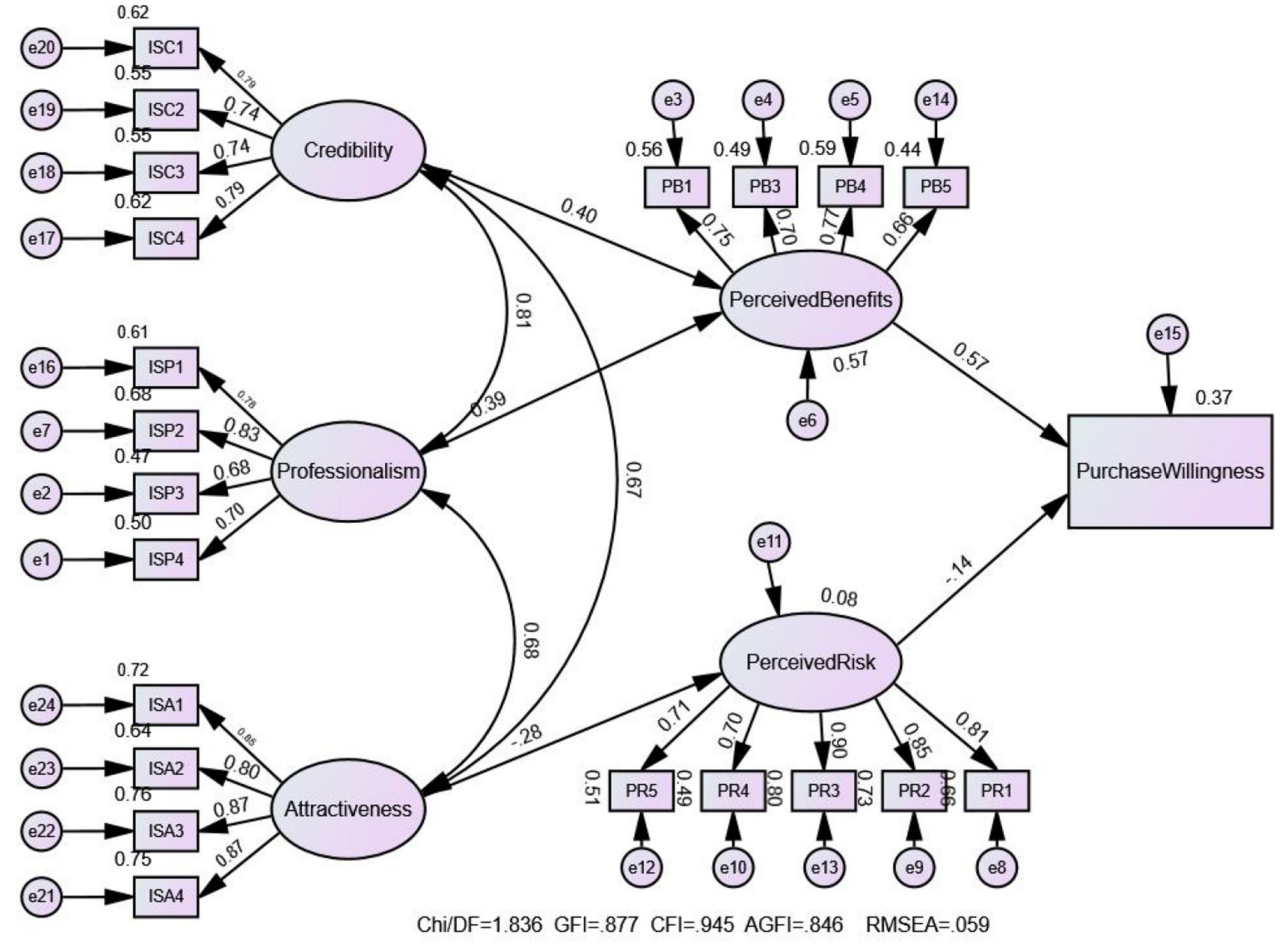
Structural equation model analysis results.

The results of the model analysis are shown in Figure 2, there is no structural relationship between the credibility, professionalism and attractiveness of local governments, but there is a correlation between them. Table 3 shows that there is a correlation between them and there is good discriminant validity, so the model construction is reasonable. The regression paths that passed the significance test are marked in the figure, and the paths that did not pass the significance test have been removed.

### 5.3. Mediating effect test

In this paper, we used AMOS28.0 software to test the effect of perceived benefit and perceived risk as concurrent mediating variables based on the Bootstrap procedure. In total of 2,000 Bootstrap samples were randomly repeated within the original sample with a 95% confidence interval, and the results are shown in Table 6.

**Table 6:** Bootstrap estimation results of mediating effects.

**Paths**	**Effect value**	**Standard error (SE)**	**Bias-corrected 95% confidence interval**
**Direct effect**			
Credibility–>purchase willingness	0.134	0.119	(−0.072, 0.393)
**Indirect effects**			
Credibility –> perceived benefits –> purchase willingness	0.340	0.100	(0.209, 0.638)
Credibility –> Perceived risk –> purchase willingness	0.023	0.015	(0.002, 0.068)
**Direct effect**			
Professionalism–>purchase willingness	0.247	0.096	(0.042, 0.430)
**Indirect effects**			
Professionalism–>perceived benefits–>purchase willingness	0.354	0.116	(0.180, 0.665)
Professionalism–>perceived risk–>purchase willingness	0.026	0.017	(0.002, 0.074)
**Direct effect**			
Attractiveness–>purchase willingness	0.217	0.066	(0.070, 0.341)
**Indirect effects**			
Attractiveness–>perceived benefits –> purchase willingness	0.209	0.053	(0.141, 0.315)
Attractiveness –> perceived risk –> purchase willingness	0.028	0.017	(0.004, 0.060)

In the effect of credibility on consumption willingness, the confidence interval of the direct effect is (−0.072, 0.393), and the range includes 0, that is, the direct effect is not significant; while the confidence interval range of the indirect effect through both perceived benefit and perceived risk does not include 0, indicating that the indirect effect is significant; therefore, perceived benefit and perceived risk play a fully mediating role between credibility and purchase willingness.

In the effect of professionalism on purchase willingness, the confidence interval of the direct effect is (0.042, 0.430), the range does not include 0, that is, the direct effect is significant; and the confidence interval of the indirect effect through perceived benefit and perceived risk also does not include 0, in other words, the indirect effect is also significant; therefore, perceived benefit and perceived risk play a partially mediating role between professionalism and purchase willingness. The indirect effect of perceived benefits accounted for 56% of the total effect, while the indirect effect of perceived risks accounted for 4% of the total effect. Similarly, perceived benefit and perceived risk play a partially mediating role between attractiveness and purchase willingness. The indirect effect of perceived benefits accounted for 46% of the total effect and the indirect effect of perceived risks accounted for 6% of the total effect.

## 6. Conclusion and discussion

### 6.1. Conclusion

Based on the survey data, this paper discusses the purchase intention of low-carbon agricultural products in the context of low-carbon agricultural development by taking regional brand agricultural products as a representative of low-carbon agricultural products, empirically analyzes the influence of local government information source characteristics on consumers' purchase willingness, complements the research on building the regional brand and developing low-carbon agriculture, verifies the mediating role of perceived benefits and perceived risks, and further analyzes the types of mediating role. The empirical results show that: first, the credibility and professionalism of local governments play a positive influence on the purchase willingness of low-carbon agricultural products through perceived benefits, with the greatest influence of credibility. Second, the attractiveness of local governments plays a positive influence on consumers' purchase willingness through perceived risks. Third, perceived benefits play a fully mediating role between credibility and purchase willingness, and a partially mediating role between professionalism and purchase willingness; perceived risks play a partially mediating role between attractiveness and purchase willingness.

### 6.2. Managerial implications

Based on the above conclusions, in order to promote low-carbon emission reduction in agriculture and develop low-carbon agricultural products with regional brand agricultural products as an important way, local governments as an important source of information need to pay attention to the following aspects.

First, we need to enhance the credibility of local governments and improve the credibility of official endorsements through multiple channels. The credibility of the government needs to be guaranteed by correct leadership and efficient implementation. Local governments, as an important window for uploading and transmitting, should take advantage of the regional brands of agricultural products with good development to actively apply and promote the carbon label for agricultural products by way of pilot projects on the basis of establishing a sound carbon labeling system by the central government departments. The local government's support and supervision of low-carbon agricultural products should be fully implemented. With the local Agriculture and Rural Affairs Bureau as the leading unit, multiple government departments should be cooperated in the process of promoting the transformation of regional brand agricultural products to low-carbon agricultural products. Actively take actions to popularize low-carbon production and planting technology, accelerate the improvement of the carbon labeling system for agricultural products, supervise the quality of low-carbon agricultural products, crack down on fake and shoddy behaviors, and deliver the implementation effect to the consumers through multiple channels, establish a credible image in the minds of consumers, so as to improve consumers' perception of the value of low-carbon agricultural products and agricultural low-carbon development.

Second, we need to enhance the professionalism of local governments and improve the quality of information delivered. As one of the main leaders in the consumption of low-carbon agricultural products, it is crucial for the information delivered by local governments to the public. It is necessary to properly strengthen the professionalism of the information content, focusing on the carbon emission standards, quality identification, quality control and other aspects of low-carbon agricultural products, so as to improve consumers' perception of the quality of low-carbon agricultural products.

Third, we need to enhance the attractiveness of local governments and create an approachable government image. The plates related to low-carbon agriculture in the official platform can be sorted out and integrated into relevant topics according to the consumers' attention. The information about greenhouse gas emissions of the whole life cycle of agricultural products can be made more visible, readable, and perceptible by means of graphics, short videos, and live broadcasts. The frequency of information release can be effectively controlled in order to increase the consumers' acceptance of local government through high-quality information, so as to enhance the efficiency of the information released by the government to reach consumers.

Fourth, we need to enhance the perceived value of consumers by uniting multiple entities. In addition to local governments, agricultural industry associations, leading enterprises and cooperatives are all important subjects for the promotion of low-carbon agricultural products. The government should unite various forces to upgrade regional brand agricultural products to low-carbon agricultural products, and constantly enhance consumers' recognition of the quality and value of low-carbon agricultural products through diversified and multi-channel publicity methods. The media is also an important player. Local governments should guide the media to help promote and publicize the development of carbon labeling of agricultural products, and convey the concept of low-carbon agriculture and green consumption to the whole society. The basic rights of consumers should be protected to reduce risks in the purchase process, so as to support the development of low-carbon agriculture from the consumer side.

### 6.3. Theoretical contributions and research limitations

There is a marginal contribution in this paper in terms of research perspective. This paper takes the local government, which plays an important role in the development of low-carbon agricultural products, as the information source, focuses on the important role of local government in the promotion of low-carbon agricultural products, analyzes the influence of local government information sources on consumers' purchase willingness, provides diversified thinking paths for the development of low-carbon agricultural products, and expands the research on the consumer side of low-carbon agriculture.

There are a few shortcomings in this paper. For example, at present, the practice and research of low-carbon agricultural products in China are in the initial time. This paper only discusses the potential and possibility of developing regional brand agricultural products into low-carbon agricultural products from the literature level, but does not build indicators to quantify the potential from the perspective of empirical analysis, and does not show the contribution degree of regional brand agricultural products to the development of low-carbon agriculture through data.

## Data availability statement

The raw data supporting the conclusions of this article will be made available by the authors, without undue reservation.

## Author contributions

Conception and design of study and drafting the manuscript: PX and LW. Acquisition of data: LW, PX, ML, and YL. Analysis of data: LW. Revising the manuscript critically for important intellectual content: PX, ML, and YL. All authors contributed to the article and approved the submitted version.
